# Integrative analysis of T cell-associated markers in Ewing sarcoma reveals prognostic signatures and immune dynamics

**DOI:** 10.3389/fimmu.2025.1586544

**Published:** 2025-06-18

**Authors:** Umair Ali Khan Saddozai, Chenxu Liu, Fei Yan, Zhendong Lu, Muhammad Babar Khawar, Muhammad Usman Akbar, Saadullah Khattak, Haibo Sun, Ping Yang

**Affiliations:** ^1^ Institute of Translational Medicine, Medical College, Yangzhou University, Yangzhou, Jiangsu, China; ^2^ Jiangsu Key Laboratory of Experimental and, Translational Non-Coding RNA Research, Yangzhou, China; ^3^ Department of Oncology, The Eighth People’s Hospital of Shanghai, Shanghai, China; ^4^ Department of Medical Oncology, Beijing Tuberculosis and Thoracic Tumor Research Institute, Beijing Chest Hospital, Capital Medical University, Beijing, China; ^5^ Oujiang Laboratory; Key Laboratory of Alzheimer's Disease of Zhejiang Province, Institute of Aging, Wenzhou Medical University, Wenzhou, China; ^6^ Department of Preventive Medicine, Institute of Biomedical Informatics, Bioinformatics Center, Henan Provincial Engineering Center for Tumor Molecular Medicine, School of Basic Medical Sciences, Henan University, Kaifeng, Henan, China

**Keywords:** Ewing sarcoma, T cell associated genes, immune infiltration, prognostic signature, proliferation

## Abstract

**Background:**

Ewing sarcoma (ES) is a rare and aggressive pediatric bone malignancy with poor prognosis, driven by therapy-resistant tumor microenvironments (TME). The TME plays a critical role in tumor progression through a complex and dynamic network of reciprocal interactions among immune cells (dysfunctional T cells, immunosuppressive macrophages), stromal components (cancer-associated fibroblasts), and tumor cells. These interactions collectively shape the immune landscape, promote immune evasion, and contribute to therapeutic resistance. Identifying reliable prognostic markers remains a critical challenge.

**Methods:**

Here we performed an integrated single-cell RNA sequencing, WGCNA, and bulk RNA-seq analyses to investigate tumor-immune interactions. Differentially expressed genes (DEGs) intersected with T cell markers identified a total of 174 T cell-associated genes. Functional enrichment analysis and molecular subtyping were performed to explore immune-related pathways. A prognostic model based on *CLEC11A*, *BDP1*, and *ID3* was constructed using Cox regression and validated in external datasets. Immune infiltration was assessed using the CIBERSORT algorithm.

**Results:**

T cell marker analyses revealed key roles in pathways such as PI3K-Akt signaling and immune modulation. Molecular subtyping identified two clusters with distinct immune microenvironments: Cluster C1 (immunosuppressive phenotype and poorer prognosis) and Cluster C2 (functionally active immune profile associated with better prognosis). The prognostic model demonstrated high predictive accuracy for 1-, 3-, and 5-year survival (AUC: 0.85, 0.82, 0.78). Additionally, a higher tumor mutation burden (TMB) with low survival rate has been observed in High-risk group. Immune infiltration analysis showed higher CD8+ T cell and dendritic cell activity and immune checkpoint expression in low-risk groups. Experimental validation demonstrated that ID3 silencing inhibited tumor cell proliferation and induced cell cycle arrest in ES cell lines.

**Conclusion:**

Together, our study identified *CLEC11A*, *BDP1*, and *ID3* as key T cell associated prognostic markers and developed a validated model to predict survival outcomes in ES. Insights into T cell markers and tumor-immune dynamics offer promising advances in prognostic assessment and immunotherapy for ES. Furthermore, the role of ID3 in immune evasion and tumor proliferation underscores its potential as a therapeutic target, providing new avenues for immune checkpoint regulation and personalized treatment strategies.

## Introduction

Ewing sarcoma (ES) is a bone and soft tissue malignant pediatric tumor. Although a significant progress has been made in therapeutic approaches in surgery, chemotherapy and radiation over the past four decades but the five-year survival rate is still less than 30% with recurrent metastasis (1). This therapy resistance is driven by tumor microenvironment (TME) which highlights the urgent need of new therapeutic paradigms to represent unmet need (2, 3). TME plays a critical role in influencing tumor behavior and therapeutic outcomes.

Single-cell analysis has revolutionized the understanding of tumor heterogeneity, offering detailed insights into the cellular composition and immune dynamics within tumors (3–7). Using single-cell sequencing data, researchers can model cell differentiation trajectories and identify genes associated with specific differentiation stages, creating opportunities to uncover previously unknown differentiation-related genes. Furthermore, weighted gene co-expression network analysis (WGCNA) provides a powerful approach which can analyze the expression of genes pattern in multiple samples. WGCNA generates different modules by clustering of genes with same expression profile, it facilitates the exploration of correlations between these modules and phenotypic characteristics such as tumor grade (8).

Although immunotherapy has achieved significant success in hematologic cancers, its effectiveness in solid tumors such as in ES has been limited (9, 10). This limitation is largely attributed to the complex and immunosuppressive nature of the TME, which consists of tumor cells, immune cells, and stromal cells interacting via a cascade of cytokine and chemokine signaling (11, 12). These interactions regulate tumor progression and contribute to therapy resistance. Dysfunction in both innate and adaptive immune responses further exacerbates the immunosuppressive nature of the TME, enabling tumors to evade immune surveillance (13, 14).

T cells are the component of adaptive immune response that particularly plays a significant role in ES (3, 15). Though, prolonged exposure to tumor antigens often leads to T cell dysfunction, compromising their ability to execute effective antitumor responses (16). Emerging evidence suggests that the regulatory mechanisms underlying T cells disfunction are extremely conserved, indicating common genetic pathways may drive this dysfunction across different tumor types, despite tumor-specific variations (17). Considering the pivotal role of T cells in ES and their contribution to immune evasion, it is crucial to conduct a comprehensive investigation into the combined effects of T cell marker genes in ES.

## Materials and methods

### Identification of T cell associated gene using single-cell RNA sequencing data analysis

A dataset of GSE243347 was used to perform single-cell profiles analysis. To ensure high-quality single-cell RNA sequencing (scRNA-seq) data, a rigorous filtering process was applied to the raw data matrix for each cell. Genes detected in fewer than five cells were excluded from the analysis. Additionally, cells expressing fewer than 100 genes or exhibiting mitochondrial gene expression levels exceeding 5% were also removed to ensure data quality Data preprocessing and downstream analyses were performed using the Seurat R package (18). Normalization was conducted using the normalize data function with a scale factor of 10,000, employing the “log normalize” method. The “find variable features” function was utilized to identify the 1,500 most variable genes. Dimensionality reduction was performed via principal component analysis (PCA) using the Run PCA function, with significant principal components (PCs) determined through the Jack Straw method, based on the proportion of variance explained. Cell clustering was performed using the “find neighbors” and “find clusters” functions under default parameters, while visualization of cell clusters was achieved with t-distributed stochastic neighbor embedding (t-SNE) using the Runts NE function. DEGs across cell types were identified using the “find all markers” function, applying stringent thresholds for significance (adjusted P-value < 0.05) and effect size (|log2(fold change) | > 1). Cluster annotation was conducted through a reference-based approach, leveraging data from the human primary cell atlas (19) and the single R tool (20) to refine and validate cluster identities. These comprehensive steps ensured robust data preprocessing, high-quality clustering, and biologically meaningful interpretation of the scRNA-seq datasets.

### Differential gene analysis

The microarray datasets GSE17679, GSE45544, GSE68776, and GSE142162 were obtained from the NCBI GEO database (www.ncbi.nlm.nih.gov/geo/) and generated using the GPL6244, GPL5175, GPL16311 and GPL570 platforms. To maintain platform uniformity, 20 samples from the GSE37371 dataset (analyzed on the GPL96 platform) were excluded. For further analysis, normalized microarray data from 22 normal muscle samples and 479 tumor samples were used. The raw data (CEL files) were normalized using the “Affy tool (version 1.68.0)” in R software (version 4.0.3). Probe-level data were annotated using microarray annotation files, and for genes with multiple probes, the expression values were averaged, with the highest expression value retained for each gene. Overlapping genes across different platforms were aligned to create a unified dataset. To address batch effects and ensure dataset consistency, the “Limma package (version 3.46.0)” was employed. DEGs between tumor and normal tissues were identified based on the statistical thresholds of |logFC| > 1 and adjusted P-value < 0.05.

### Weighted correlation network analysis

The combined dataset was then subjected to WGCNA. The analysis of the structure and function of gene regulatory networks is made possible by WGCNA, a potent analytical technique that helps to extract significant insights from high-throughput gene expression data (8). Identifying gene modules, finding possible biomarkers, connecting gene modules to clinical traits, carrying out functional enrichment analysis, and building gene regulatory networks are the main application of WGCNA.

### Screening and functional annotation of T cell-related genes

For T cell-associated markers, 174 differentially expressed T cell marker genes were identified through an intersection analysis of DEGs and T cell marker genes, integrating single-cell, WGCNA, and bulk RNA-seq data. The limma R package was used to identify T cell-related DEGs, with both upregulated and downregulated genes selected based on the criteria of adjusted p-value < 0.05 and |log2FC| > 1. To explore the functional implications, enrichment analyses were performed using the Kyoto encyclopedia of genes and genomes (KEGG) and gene ontology (GO) databases, facilitated by the ClusterProfiler R package. Statistical significance for the enrichment analysis was set at adjusted p-value < 0.05, providing insights into the biological roles of these marker genes.

### Subtype evaluation

T cell associated marker obtained by using the intersection between DEGs from GEO differential expression analysis and T cell marker genes from single-cell data were used to identify subtypes. The “Consensus Cluster Plus” R program identified the molecular subtypes of ES. Sub-cluster prognostic differences were assessed using Kaplan–Meier (K–M) analysis. Heatmap was used to illustrate the connections between subtypes and clinical traits by employing chi-square tests.

### Prognostic signature establishment and verification using T cell associated genes

For the development of a prognostic signature, we utilized the ICGC2 dataset comprising 57 ES samples. Independent genes associated with overall survival (OS) were identified through multivariate Cox regression analysis using the “coxph” function from the “survival” R package. This analysis facilitated the construction of a prognostic model, with gene coefficients recorded for further reference. The model was established by combining gene mRNA expression levels with their corresponding risk coefficients using the formula: Risk score = coefficient × expression (mRNA). Based on the median risk score, patients were categorized into low- and high-risk groups. The predictive performance of the model was evaluated using the area under the curve (AUC) obtained from the “survival ROC” package, while Kaplan-Meier (K-M) analysis was employed for survival analysis. The prognostic model was further validated using external cohorts from the GSE63157 and GSE17674 datasets. Cox regression analysis confirmed the signature as an independent risk factor. Stratified analysis and nomogram construction were performed following correlation analyses between clinical characteristics and risk scores. Calibration plots were generated to compare the predicted 1-, 3-, and 5-year mortality rates with actual outcomes, ensuring the model’s reliability.

### Immune-related analysis in risk groups

To further explore the relationship between high- and low-risk groups, immune-related analyses were conducted. The CIBERSORT algorithm was employed to determine the composition of immune cell populations within each ES sample. Single-sample gene set enrichment analysis (ssGSEA) was used to assess immune cell activity and immune functions across the samples. Mutation analysis was performed using the “maftools” R package, and the tumor mutation burden (TMB) was calculated and compared between the high- and low-risk groups. Additionally, survival analysis was conducted to evaluate the association between TMB scores and patient outcomes.

### Chemotherapy sensitivity prediction

To assess the potential of the T cell-associated gene score as a predictive biomarker for chemotherapeutic response in ES patients, its association with drug sensitivity was analyzed. The half-maximal inhibitory concentration (IC50) values of commonly used chemotherapeutic agents were estimated using the “pRRophetic” R package. This method enabled the evaluation of the gene signature’s predictive ability in determining chemotherapy sensitivity.

### Cell lines and culture

Human ES cell lines A673 (Wuhan Pricella Biotechnology CO., Ltd.) and RD-ES (Meisen Chinese Tissue Culture Collections) were cultured in DMEM or RPMI-1640 medium at 37°C in a humidified environment with 5% CO_2_.

### ID3 knock down with siRNA

siRNAs were procured from GenePharma (Suzhou, China). Gene knockdown was performed using siRNA specifically targeting ID3. A stable non-specific siRNA (siNC) served as the negative control. Transfection was carried out using the Lipo8000 kit in accordance with the manufacturer’s protocol. Cells were harvested 48 hours post-transfection for subsequent analysis.

### Real-time quantitative reverse transcription PCR

Total RNA was extracted using VeZol Reagent (Nanjing Vazyme Biotech Co., Ltd). Purified RNA was reverse-transcribed into cDNA using HiScript II Q RT SuperMix for qPCR (Nanjing Vazyme Biotech Co., Ltd). GAPDH was used as an internal control. All procedures were performed in accordance with the manufacturer’s instructions.

### Cell proliferation assay

The cell proliferation assay was performed by seeding transfected cells at a density of 1 × 10^5^ cells per well in a 24-well plate. Cell counts were assessed under a microscope at 0, 24, 48-, 72-, 96-, and 120-hours post-seeding to evaluate proliferation dynamics over time.

### Flow cytometry for cell cycle distribution analysis

48 hours after transfection, cells were harvested, fixed overnight in 70% ethanol at 4°C, treated with RNase, and then stained with propidium iodide (PI) in the dark for 30 minutes for cell cycle analysis. CytoFLEX Flow cytometer (Beckman Coulter, Inc.) was used to determine the distribution ratio of each phase of the cell cycle.

### Statistical analysis

Statistical analyses were performed using SPSS v.19.0 software (SPSS; Chicago, IL, USA). Differences between two groups were assessed using independent samples t-tests. Differences among three or more groups were analyzed using one-way analysis of variance (ANOVA). All data are expressed as mean ± standard deviation. A P-value < 0.05 was considered statistically significant.

## Results

### Identification of T cell associated genes expression profiles

**Figure 1:**
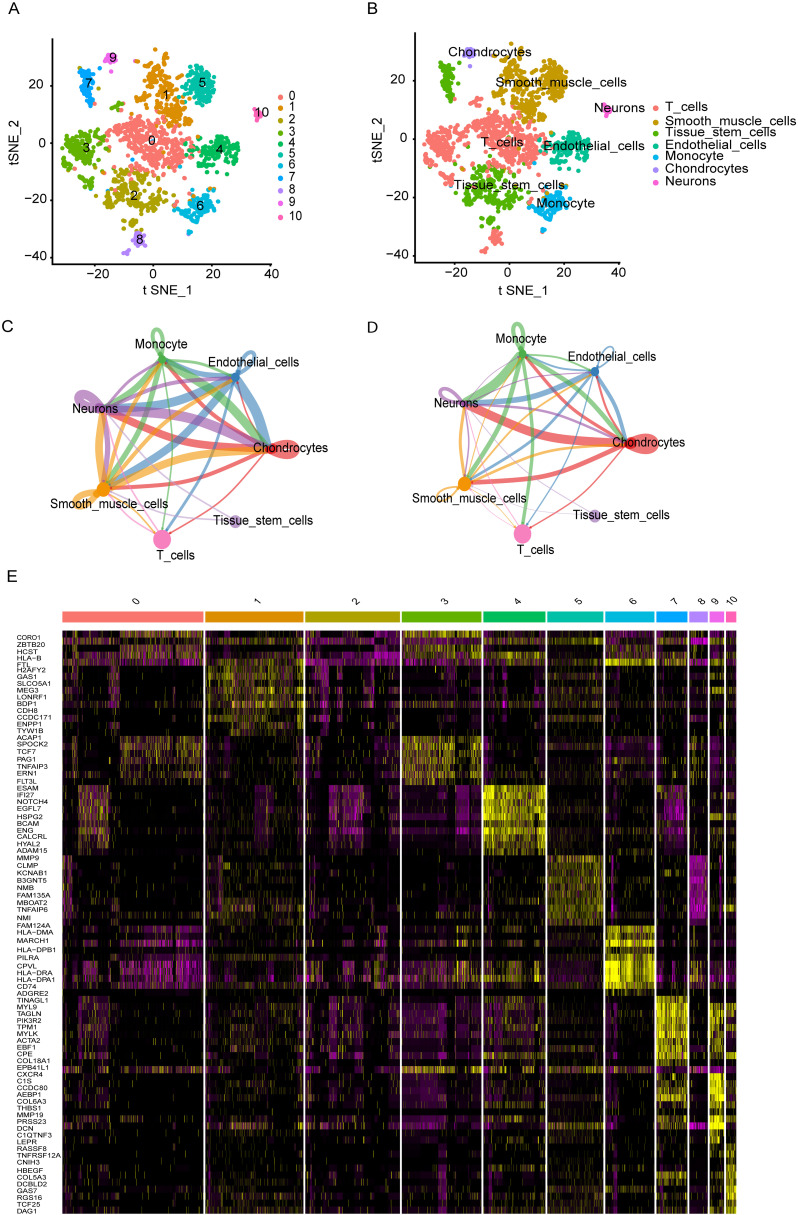
Single-cell transcriptomic analysis reveals cellular heterogeneity and intercellular interactions **(A, B)** t-SNE plots identified various display distinct cell clusters. **(C)** Number of ligand-receptor interactions between cell types. T cells show moderate connectivity, interacting most with neurons, chondrocytes, and tissue stem cells. **(D)** Mean interaction strength between cell types. T cells, despite fewer interactions, exhibit high signaling intensity with smooth muscle cells and monocytes, suggesting targeted, functional crosstalk. **(E)** A heatmap highlights differentially expressed genes across cell populations, revealing key molecular signatures that may contribute to cell-type-specific functions and interactions.

Based on single-cell profiles from GSE243347, which included 27 samples, we derived a gene expression matrix encompassing 7,182 cells and 10,128 features for further analysis. Using the Harmony algorithm for batch effect correction and dimensionality reduction, we identified 10 distinct clusters (Cluster 0-10) through unsupervised analysis of single-cell transcriptomic data (**Figure 1A**). These clusters were defined based on transcriptional similarity and visualized using t-SNE projection. To assign biological identity, we annotated the clusters using established marker genes. The resulting clusters were categorized into seven major cell types: T cells, smooth muscle cells, tissue stem cells, endothelial cells, monocyte, chondrocytes and neurons. T cells are represented by the red-colored cluster (**Figure 1B**). Notably, the distribution of T cells varied significantly across ES patient samples, leading to the identification of 489 ES-associated T cell marker genes (**Supplementary Table S1**). This diverse cellular landscape underscores the complexity of the tissue microenvironment. The “CellChat” approach revealed substantial connectivity between different cell types, particularly among T cells, macrophages, and endothelial cells, which exhibited high interaction strength and a large number of interactions. The number of interactions, representing how many distinct ligand–receptor pairs exist between cell types. In this context, T cells showed notable but comparatively fewer interactions than highly connected cell types such as neurons and chondrocytes, which formed the most extensive communication network in terms of interaction count (**Figure 1C**). In contrast, the weights of interactions, defined as the mean interaction strength, which reflects the average signaling intensity between cell types. Here, T cells exhibited strong signaling activity, particularly with smooth muscle cells and monocytes, despite having fewer total interactions. This suggests that T cells may engage in functionally potent and selective signaling, indicating a targeted regulatory role within the tumor microenvironment (**Figure 1D**). Furthermore, DEG analysis also identified the several genes specific to the cell populations (**Figure 1E**).

### Identification of T cell associated genes and biological enrichment functions

**Figure 2:**
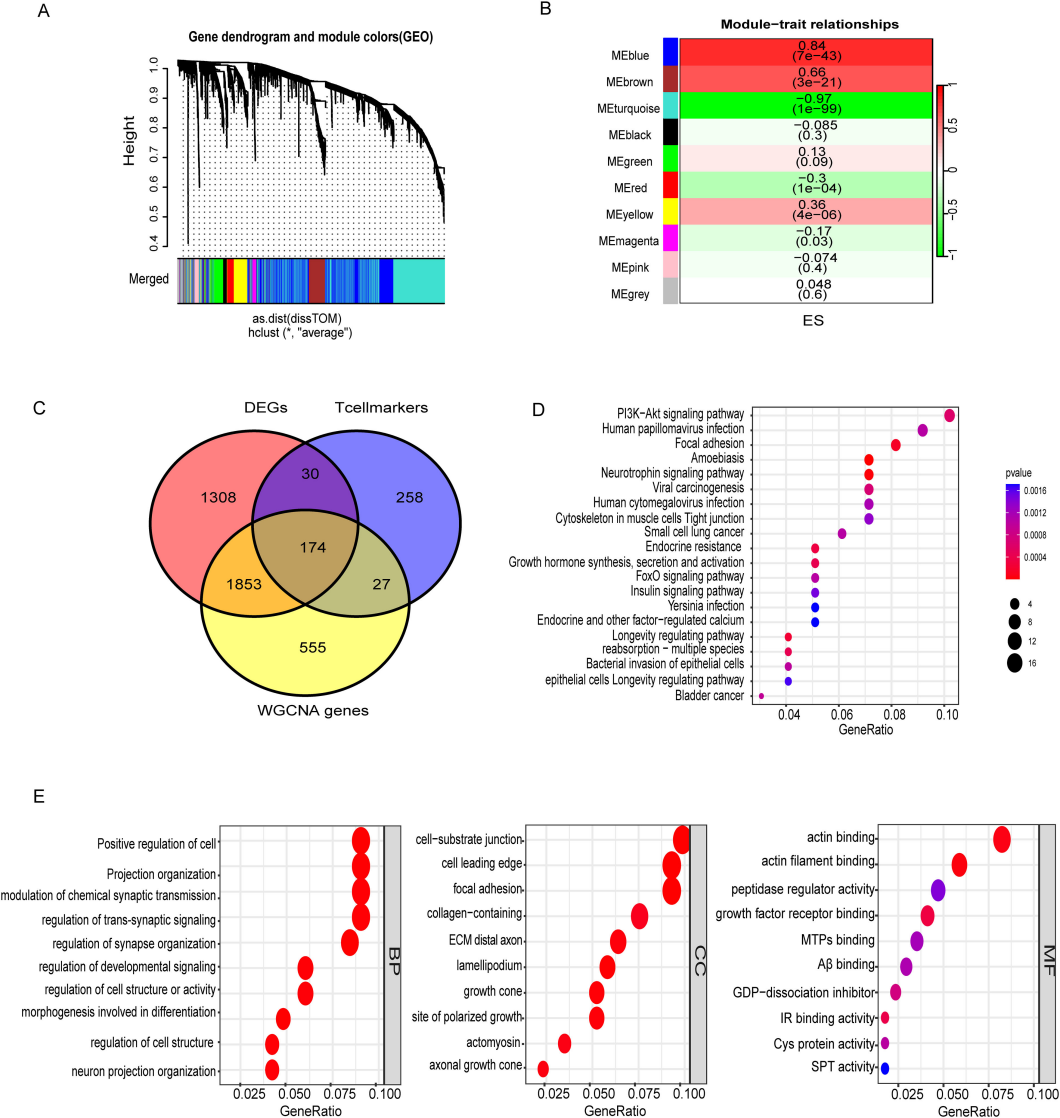
Identification of T cells associated genes and enriched pathways. **(A)** Gene dendrogram with module colors from the GEO dataset. **(B)** Module-trait relationships showing correlations and significance. **(C)** Overlap of DEGs, T cell markers, and WGCNA-identified genes, emphasizing immune and structural gene associations. **(D)** KEGG pathway analysis highlighting PI3K-Akt signaling, focal adhesion, and viral carcinogenesis. **(E)** GO enrichment of biological processes, cellular components, and molecular functions. Color represents the adjusted p-value (red = higher significance; blue = lower significance). Dot size reflects the gene ratio (proportion of genes enriched in each pathway).

Comparisons between tumor and healthy muscle tissues from the NCBI database identified 3,365 DEGs (**Supplementary Table S2**). WGCNA analysis revealed distinct module-trait relationships in ES tumors, as depicted in the gene dendrogram and module colors from the GEO merged dataset (**Figure 2A**). Among these, the MEblue module showed a strong positive correlation with tumor traits (r = 0.84, p < 7e−43), whereas the Meturquoise module exhibited a robust negative correlation (r = −0.97, p < 1e−99) (**Figure 2B**). These findings suggest that these modules contain genes likely involved in key biological functions and disease mechanisms. We merged the Meblue and Meturquoise module genes and acquired the 2,609 genes by WGCNA. (**Supplementary Tables S3, S4**). The intersection of DEGs, WGCNA genes, with T cell marker genes yielded 174 differential T cell marker genes for downstream analyses (**Figure 2C**, **Supplementary Table S5**).

Pathway enrichment analysis (KEGG) revealed the involvement of these genes in critical cancer-related pathways, such as those implicated in bladder cancer, small cell lung cancer, and viral carcinogenesis, highlighting their potential roles in tumorigenesis and immune evasion. Signal transduction pathways, including PI3K-Akt and FoxO signaling, are well-established mediators of cell survival, proliferation, and apoptosis, and are often dysregulated in cancer (**Supplementary Table S6**). Moreover, pathways linked to infectious diseases, such as human cytomegalovirus infection and Yersinia infection, point to the dual roles of these genes in infection response and inflammation. GO analysis of biological processes reveals critical insights into the functional roles of these cells. In the BP category, key processes such as the positive regulation of cell projection organization, modulation of chemical synaptic transmission, and neuron projection organization were enriched, indicating roles in neural connectivity and signaling pathways. The CC analysis highlighted critical structural components, including the cell-substrate junction, focal adhesion, and collagen-containing extracellular matrix, underscoring their significance in maintaining cellular architecture and mediating cell-extracellular matrix interactions. In the MF category, enriched functions such as actin binding, insulin receptor binding, and protein-cysteine activity suggest involvement in cytoskeletal remodeling, signaling, and metabolic regulation (**Figure 2E**, **Supplementary Table S7**). DEGs within the WGCNA (MEblue and MEturquoise) module were significantly associated with T cell markers, suggesting their potential involvement in modulating immune cell functions such as antigen recognition and effector responses. These results collectively reveal the interplay between genetic regulation, immune function, and disease processes, offering a comprehensive understanding of the biological significance of these gene clusters.

### Molecular subtypes association with tumor immunological grades

**Figure 3:**
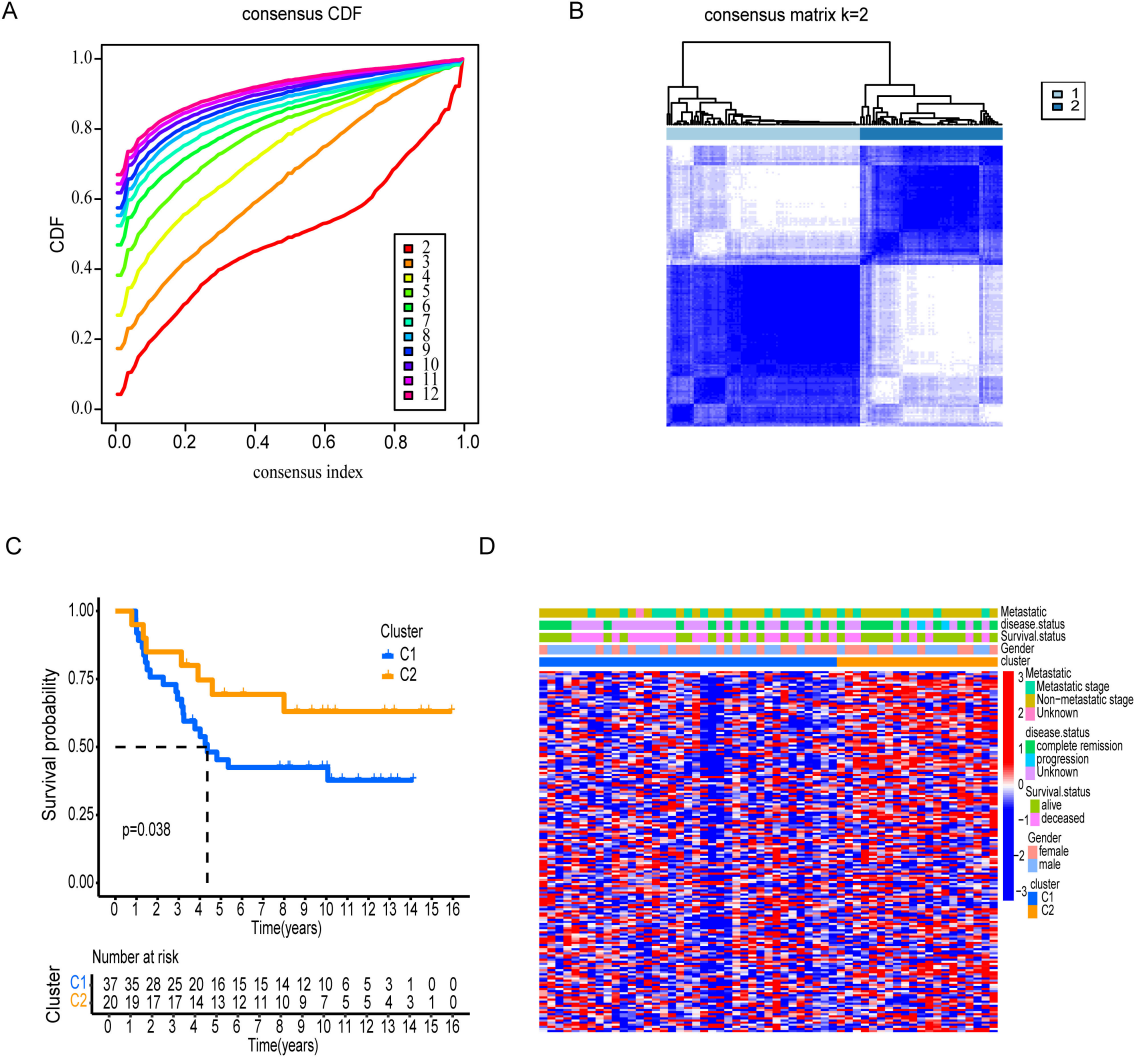
Consensus clustering and survival analysis of patient subgroups. **(A)** Cumulative distribution function (CDF) and consensus index plots validating cluster stability. **(B)** Clear distribution revealed by Consensus matrix for k=2 between two clusters. **(C)** Kaplan-Meier survival analysis comparing Clusters, C1 and C2. **(D)** Clinical feature distribution across clusters, including gender, survival status, disease status, and metastatic stage.

**Figure 4:**
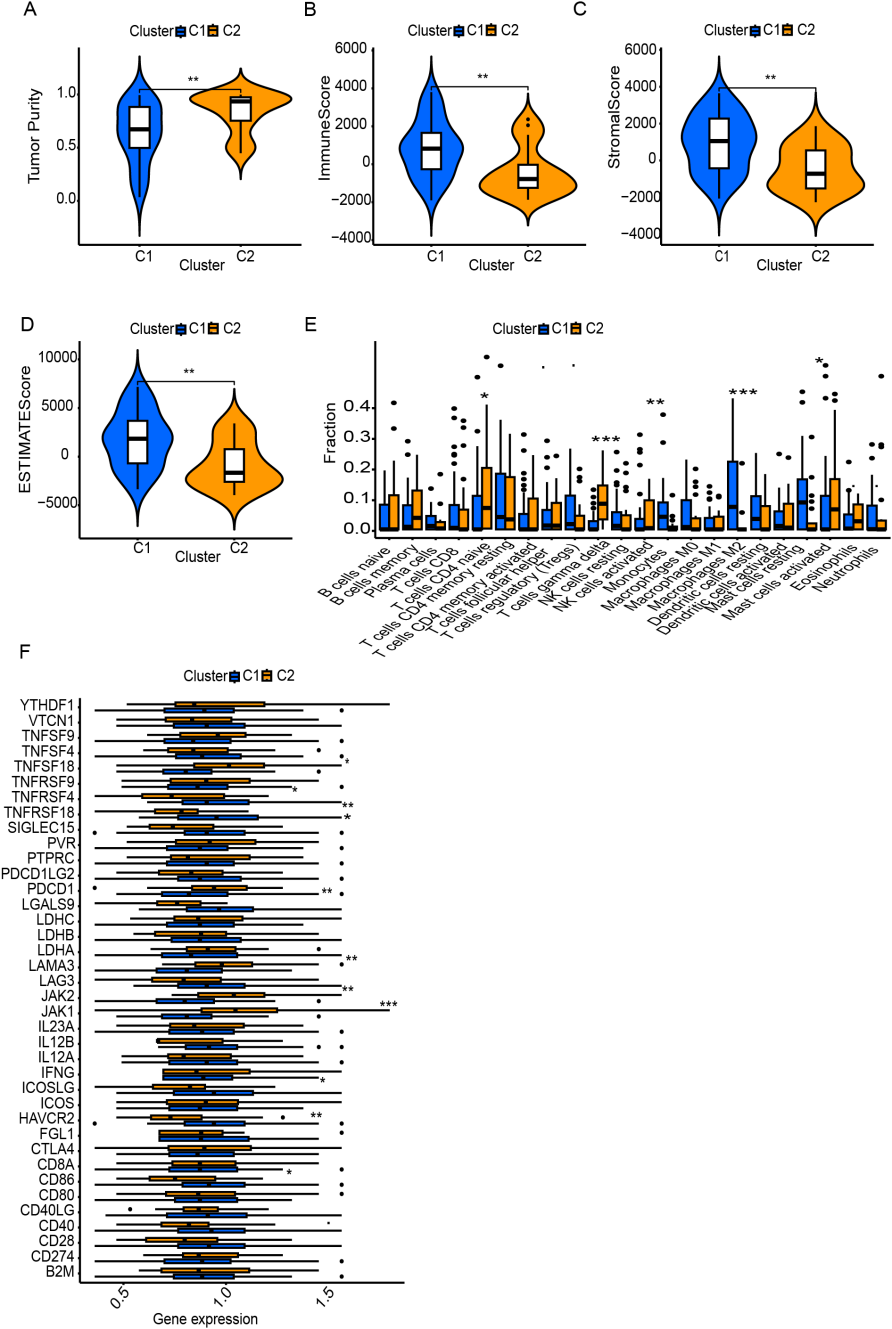
Tumor microenvironment (TME) and immune landscape differences between clusters. **(A)** Tumor purity differs significantly between C1 and C2. **(B–D)** ESTIMATE analysis shows higher immune and stromal scores in one cluster. **(E)** Immune cell infiltration analysis reveals distinct TME profiles between clusters. **(F)** Immune-related gene expression varies, particularly in checkpoint molecules and regulators. * Symbols denotes, A single * denotes a p-value of less than 0.05, indicating a statistically significant difference. Two ** represent a p-value of less than 0.01, suggesting a highly significant difference, while three *** indicate a p-value of less than 0.001, reflecting a very highly significant difference.

Using DEGs of T cell marker genes, we performed molecular subtyping, optimally clustering ES patients into two subgroups with high internal coherence (**Figures 3A, B**). Survival analysis indicates that patients in cluster C2 have a significantly better prognosis compared to those in cluster C1 (p < 0.038) (**Figure 3C**). The heatmap differentiates the distinct clusters, C1 and C2, based on their clinical features and expression profiles. Cluster C1, which includes a larger number of patients, is predominantly associated with metastatic disease, disease progression, and poor survival outcomes, indicating its potential link to an aggressive disease phenotype. In contrast, cluster C2 patients have non-metastatic and remission statuses, suggesting a less severe disease profile (**Figure 3D**). The analysis highlights distinct tumor biology and immune microenvironments between clusters C1 and C2. Moreover, Cluster C2 exhibited significantly higher tumor purity, indicating a denser tumor cell population (**Figure 4A**). In contrast, Cluster C1 showed significantly higher Immune Score, Stromal Score, and ESTIMATE Score compared to C2 (**Figures 4B–D**). Despite this elevated immune presence, Cluster C1 did not appear to mount an effective antitumor immune response. Immune cell profiling revealed that C1 was predominantly infiltrated by immunosuppressive cell populations, such as M2 macrophages and monocytes, which are known to inhibit cytotoxic immune activity and support tumor progression. Conversely, Cluster C2 displayed higher proportions of γδ T cells, CD4^+^ naïve T cells and activated mast cells, these cell types associated with innate immune activation, antitumor priming, and immune surveillance (**Figure 4E**). Similarly, at the molecular level, Cluster C1 exhibited increased expression of immunosuppressive checkpoint molecules, including LAG3 and HAVCR2 (TIM-3), both of which are canonical markers of T cell exhaustion and dysfunction. Although CD8A expression was also significantly elevated in C1, indicating the presence of cytotoxic T cells, this was likely offset by the high expression of inhibitory receptors and the presence of suppressive immune subsets, leading to functionally impaired immune responses. In contrast, Cluster C2, despite lower immune and stromal infiltration, showed significantly higher expression of IFNG, PDCD1 (PD-1), and JAK1 (**Figure 4F**). These markers are indicative of active interferon signaling, T cell receptor activation, and immune co-stimulation, suggesting that the immune cells present in C2 retain their functionality and responsiveness. Although CD8A levels were lower in C2, the expression profile supports a more immune-permissive and responsive microenvironment.

These differential gene expression patterns suggest that Cluster C1 represents an immune-infiltrated but immunosuppressive phenotype, with immune dysfunction likely driven by chronic antigen stimulation and elevated inhibitory signaling. In contrast, Cluster C2 reflects an immune-functional phenotype, characterized by more effective immune components, potentially conducive to better immunological control of tumor growth.

### Construction of T cell marker signature

**Figure 5:**
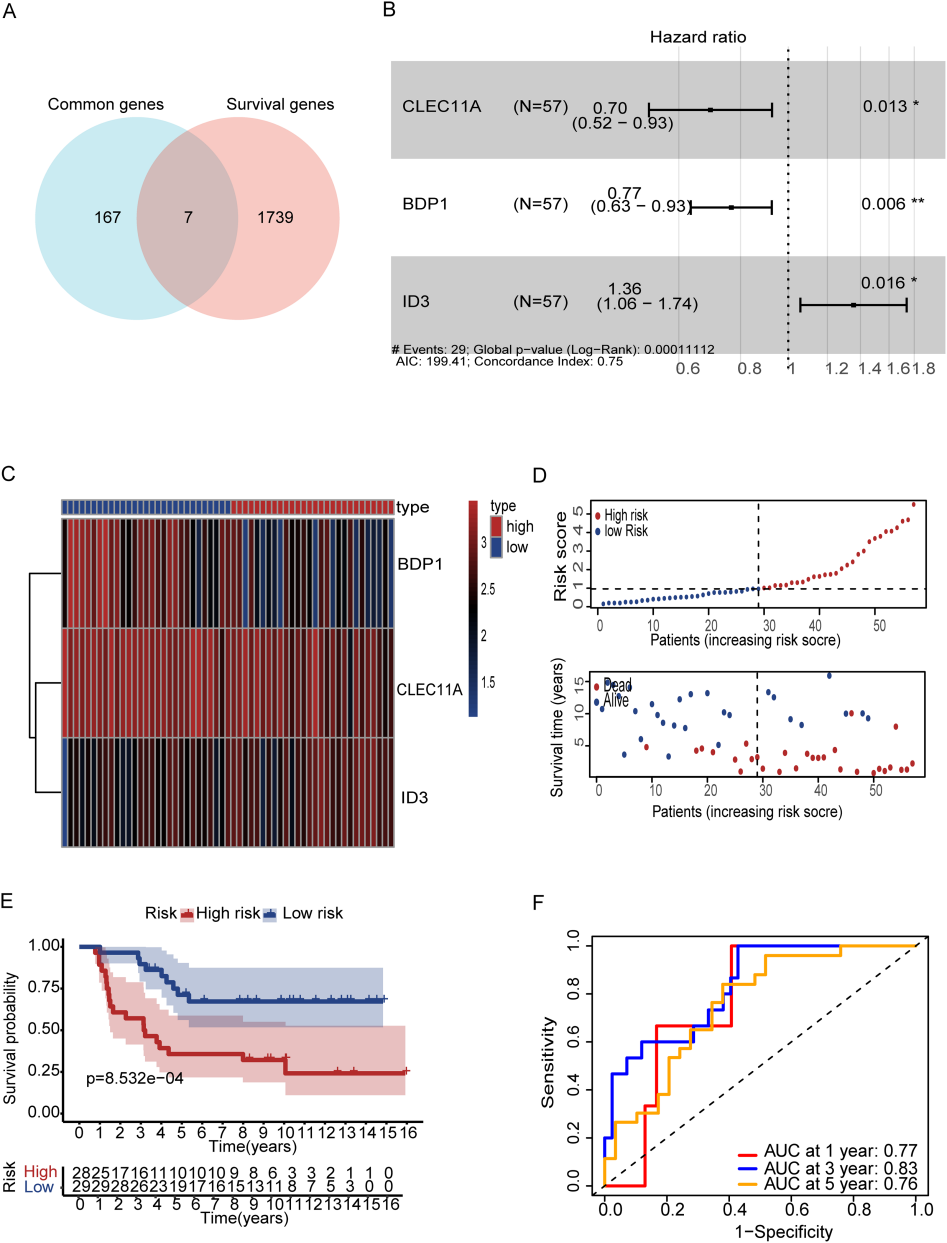
Gene based signature for prognosis analysis and risk stratification. **(A)** Vin diagram represents the genes between common and survival genes. **(B)** Forest plot showing hazard ratios (HR) for ID3, BDP1, and CLEC11A (p < 0.05). **(B)**. **(C)** Heatmap of prognostic genes **(D)** Risk score distribution correlates with survival status. **(E)** Kaplan-Meier curves reveal survival in both risk groups **(F)** ROC curves show strong predictive accuracy at 1, 3, and 5 years (AUC: 0.77, 0.83, 0.76). Symbols denotes, A single * indicates a p-value < 0.05 statistically significant, while two ** indicate a p-value < 0.01 highly significant.

**Figure 6:**
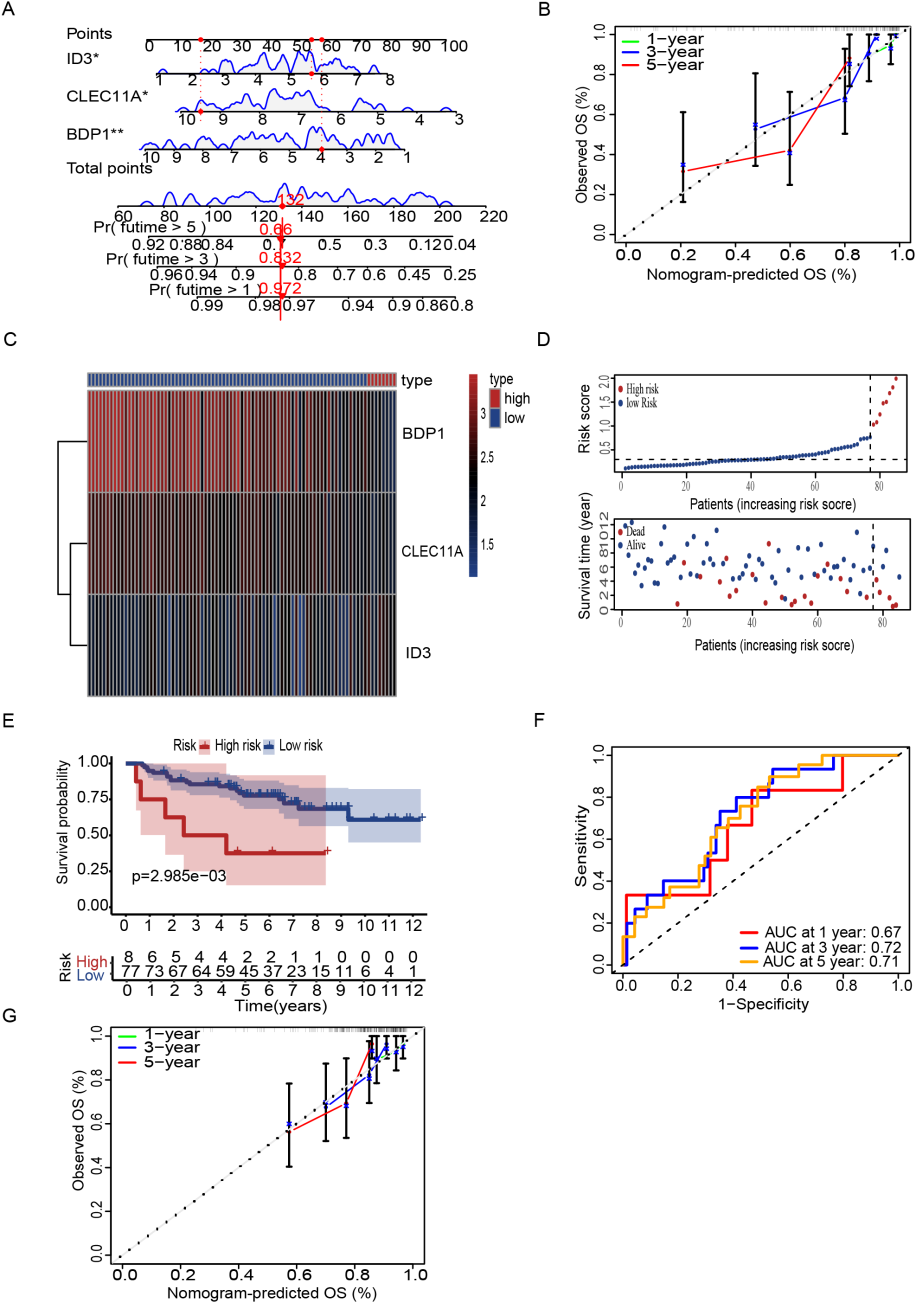
Nomogram and risk stratification for survival prediction. **(A)** Nomogram using ID3, CLEC11A, and BDP1 to predict overall survival (OS). **(B)** Calibration plots show agreement between predicted and observed OS at 1, 3, and 5 years. **(C)** Heatmap of prognostic signature genes **(D)** Risk score distribution correlates with survival status. **(E)** Kaplan-Meier curves reveal worse survival in high-risk patients. **(F)** ROC curves demonstrate predictive accuracy (AUC: 0.67, 0.72, 0.71 at 1, 3, 5 years). **(G)** Calibration plots of GSE63157 show agreement between predicted and observed OS at 1, 3, and 5 years. Symbols denotes, A single * indicates a p-value < 0.05 statistically significant, while two ** indicate a p-value < 0.01 highly significant.

To increase the specificity of candidate genes, we conducted K-M plot and univariate Cox analysis, identifying 1,746 prognostic-associated genes using the ICGC dataset, which were significantly associated with patient outcomes in the cohort (**Figure 5A**, **Supplementary Table S8**). Furthermore, by intersecting the set of these prognostic-associated genes with the set of 174 genes, we identified 7 key prognostic-associated genes crucial for subsequent analyses. We then performed a multivariate Cox regression analysis to construct the model, selecting three key genes: CLEC11A, BDP1, and ID3. The hazard ratios for these genes were calculated, and they were identified as significant in the survival analysis. CLEC11A and BDP1 were shown to be protective factors, with hazard ratios of 0.70 (95% CI: 0.52–0.93, p = 0.013) and 0.77 (95% CI: 0.63–0.93, p = 0.006), respectively, indicating that higher expression of these genes is associated with better survival outcomes. In contrast, ID3 was identified as a risk factor with a hazard ratio of 1.36 (95% CI: 1.06–1.74, p = 0.016), suggesting that its higher expression correlates with poorer survival (**Figure 5B**). Patients were stratified into high-risk and low-risk groups based on their gene expression profiles. The heatmap of gene expression for the three genes showed distinct expression patterns between high-risk and low-risk patients. The risk score distribution and survival status of patients indicated that those in the high-risk group exhibited poor survival outcomes, as shown by higher risk scores and increased mortality (**Figures 5C, D**). K-M survival curves demonstrated the significance of both risk groups (p = 5.32e-04) (**Figure 5E**). Time-dependent ROC curves were generated to assess the predictive performance of the model, achieving AUC values of 0.85 for one year, 0.82 for three years, and 0.78 for five years, indicating high predictive accuracy (**Figure 5F**). Finally, the overall survival probabilities at 1, 3, and 5 years were predicted by the nomogram using the biomarkers ID3, CLEC11A, and BDP1 expression levels, contributing to the total score. This demonstrated that patients with a high score had significantly higher probabilities of survival at 1, 3, and 5 years (**Figure 6A**). The nomogram’s performance was evaluated using a calibration curve, and the predicted OS showed a strong correlation with the survival rate. These results confirm the nomogram’s reliability and accuracy in predicting survival outcomes (**Figure 6B**).

### Validation of the T Cell Marker Signature

To further validate the prognostic performance of the model, we conducted validation by two external datasets GSE63157 and GSE17674. Subsequently, patients were stratified into high and low risk groups based on DEG T scores (**Figures 6C, D**). K-M analysis revealed that high-risk patients have low survival rate as compared to low-risk group (**Figure 6E**) and the model also exhibited a notably high AUC value in the external validation using the GSE63157 dataset (**Figure 6F**). Additionally, calibration plot also showed the strong agreement with OS at 1,3 and 5 years (**Figure 6G**). Similarly with the consistent our model shows the accuracy in other datasets of GSE17674. Patients can be observed in both high and low risk. High-risk patients showed the low survival rate a with the confirmation of AUC value of one year three years and five years, indicating the high predictive accuracy. While the calibration plots demonstrated a strong agreement with the OS at 1,3 and 5 years, further validating the reliability of the model (**Supplementary Figure S1**). The biological relevance of these findings underscores the potential of these genes as biomarkers for stratifying patients and tailoring therapeutic approaches based on genetic risk.

### Assessment of tumor immune cell infiltration and immune checkpoint pathways

**Figure 7:**
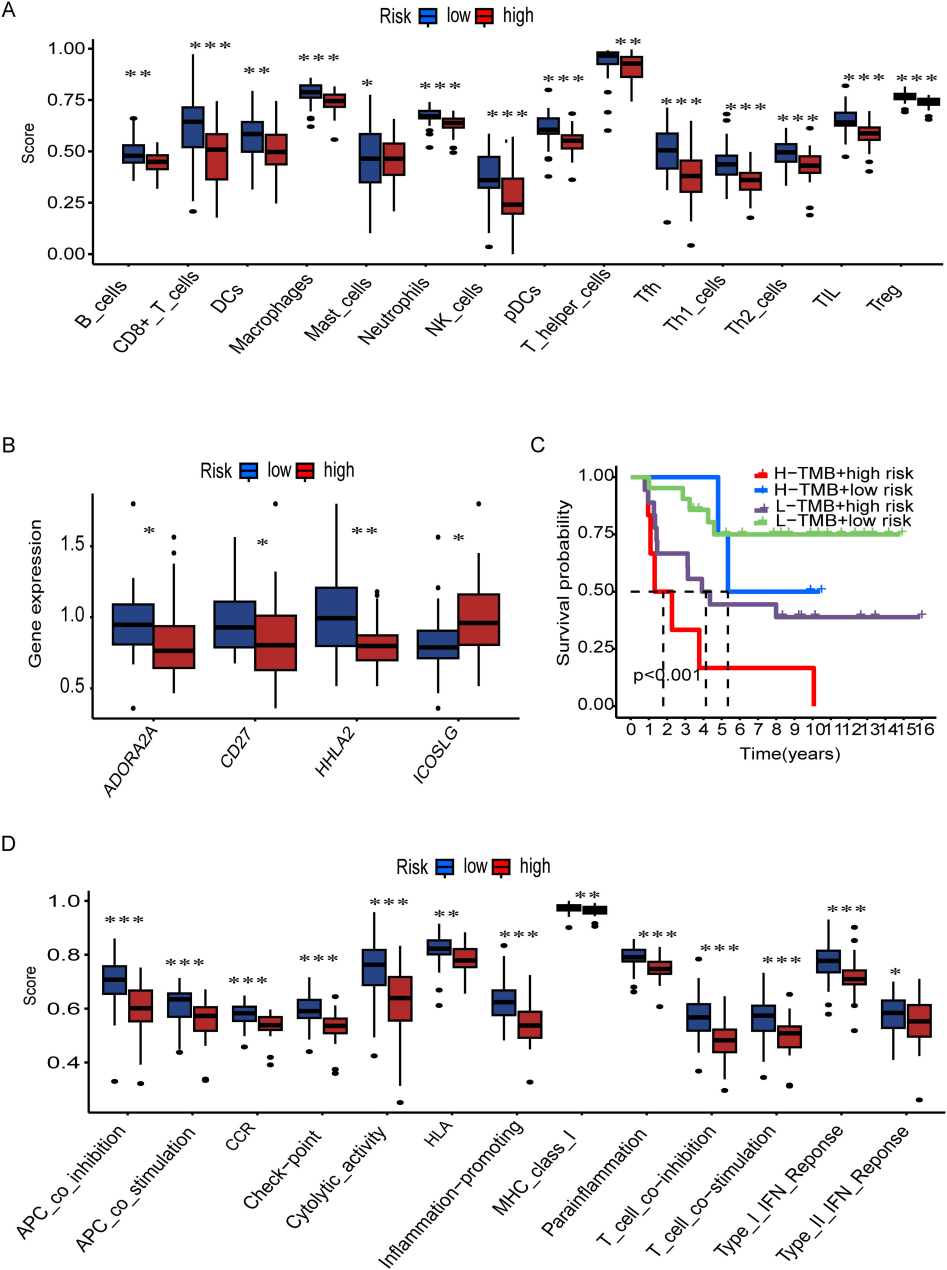
Immune landscape and survival analysis by TMB and risk groups. **(A)** Immune cell infiltration scores (e.g., B cells, CD8+ T cells) differ significantly between risk groups (***p < 0.001, **p < 0.01, *p < 0.05). **(B)** Differential expression of immune-related genes (e.g., ADORA2A, CD27) across risk groups. **(C)** Kaplan-Meier curves show high TMB + low risk correlates with better survival (p < 0.001). **(D)** Immune pathway activity (e.g., cytolytic activity, IFN response) varies by risk group.

In order to further investigate the relationship between the risk and infiltration of immune cells populations within the TME, CIBERSORT algorithm was used to compare proportion of immune cells between high risk and low risk groups. The results showed the low-risk group had higher infiltration of various immune cell population, such as B cells, CD8+ T cells, NK cells and dendritic cells show the greater prevalence of immune response (**Figure 7A**). We then investigated the potential association between risk score and the expression levels of immune checkpoint genes. Results showed that elevated level of key immune genes, including ADORA2A, CD27 and HHLA2 which have critical role in immune activity such as T cell activation and survival (**Figure 7B**). Furthermore, survival analysis starfield by TMB which reveals the high TMB with high-risk group showed the lowest survival rate between all synergistic (**Figure 7C**). In addition, specific immune function and their score such as antigen presenting cells (APC) co-inhibition, APC co-stimulation, cytolytic activity, and T cell co-stimulation are significantly higher in low-risk tumors (**Figure 7D**). This further confirms that immune functions are more active in low-risk tumors. High-risk tumors, with their lower immune cell scores and gene expression levels, may evade immune detection and response, leading to poorer clinical outcomes. Understanding these differences is vital for developing effective immunotherapies and improving cancer prognosis. Furthermore, to determine the T cell associated gene for anticipating chemotherapeutic responsiveness in ES patients. we assessed the association between our risk model and sensitivity of prevalent chemotherapeutic agents based on estimated IC50 values, which revealed significant differences between the low-risk and high-risk patient groups. Specifically, the high-risk group exhibited elevated IC50 values across the majority of chemotherapeutic agents analyzed, indicating lower predicted drug sensitivity. In contrast, the low-risk group consistently showed lower IC50 values, suggesting a higher predicted responsiveness to chemotherapy. These findings imply that patients in the low-risk group may be more likely to benefit from standard chemotherapeutic regimens, whereas patients in the high-risk group may require alternative or combination treatment strategies to achieve comparable therapeutic outcomes (**Supplementary Figure S2**).

### ID3 suppresses tumor growth and progression

**Figure 8:**
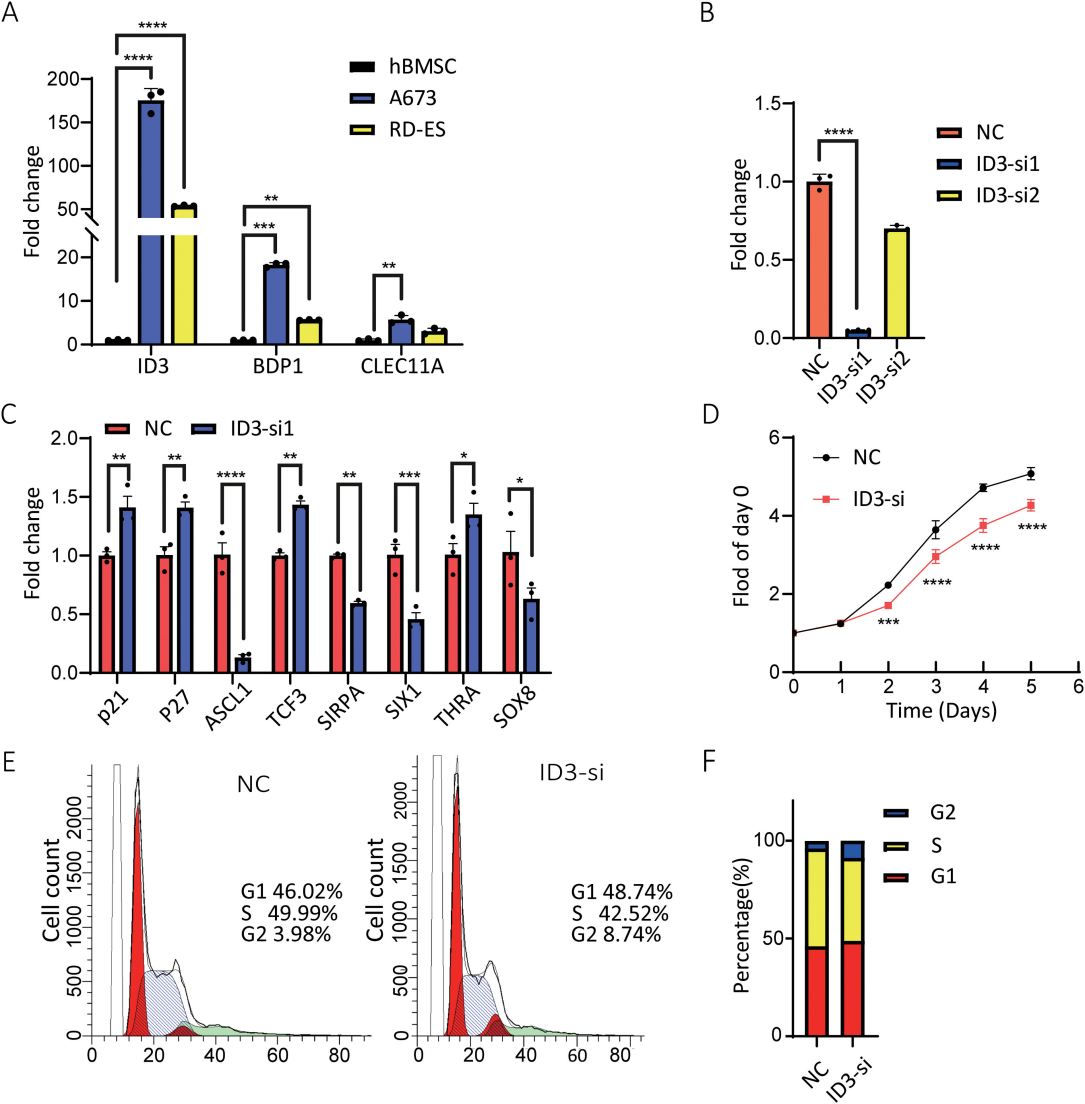
Effects of ID3 expression and silencing on A673 cells. **(A)** mRNA levels of BDP1, CLEC11A, and ID3 were higher in A673 and RD-ES cells than in hBMSC cells. **(B)** ID3 siRNA transfection reduced ID3 expression in A673 cells, with the most effective siRNA selected for further experiments. **(C)** ID3 suppression altered the expression of multiple oncogenesis-associated genes, including p21 and p27. **(D)** Proliferation analysis showed ID3 silencing reduced A673 cell growth. **(E, F)** Flow cytometry revealed an increased percentage of A673 cells in the G2 phase following ID3 knockdown. A single* indicates a p-value < 0.05 statistically significant, two ** indicate p < 0.01 highly significant, three *** indicate p < 0.001 very highly significant, and four **** indicate p < 0.0001 extremely significant.

The expression profiles of BDP1, CLEC11A, and ID3 genes in cancer cells were detected using qRT-PCR technology (**Supplementary Table S9**). Experimental data revealed that, compared to the hBMSC (control), the expression of BDP1, CLEC11A, and ID3 was significantly upregulated in the A673 and RD-ES cell lines (**Figure 8A**).

To further elucidate the biological functions of the ID3 gene in ES, RNA interference technology was employed to silence the ID3 gene in A673 cells using three specific small interfering RNAs (siRNAs) (**Supplementary Table S10**). qRT-PCR results demonstrated that one of the siRNAs exhibited the highest knockdown efficiency for ID3 and was therefore selected for subsequent experiments (**Figure 8B**). Gene expression analysis indicated that the inhibition of ID3 led to a significant increase in the expression level of the cyclin-dependent kinase (CKD) inhibitor p21, suggesting that the downregulation of ID3 could induce the upregulation of p21 in A673 cells (**Figure 8C**). Concurrently, the knockdown of ID3 also altered the expression levels of its downstream target genes, with a notable reduction in the mRNA level of SIX1 (**Figure 8C**). This finding aligns with previous research (21), which confirmed that SIX1 plays a crucial role in inhibiting cell migration, invasion, and *in vivo* metastasis, and its protein expression is regulated by EWS/FLI1. To assess the impact of ID3 knockdown on the proliferation of ES cells, the study employed a 24-hour interval cell counting method for monitoring. The results showed that, compared to the control group, ID3 knockdown significantly inhibited the proliferative capacity of A673 cells (**Figure 8D**). Flow cytometry analysis revealed that knockdown of ID3 induced G2 phase cell cycle arrest in A673 cells (**Figures 8E, F**). These experimental results collectively confirm that the gene silencing of ID3 not only suppresses the proliferative activity of A673 cells but also significantly alters their cell cycle progression.

## Discussion

Ewing sarcoma (ES) is a highly aggressive bone malignancy with an inaccurate prognosis. Its rarity, with an incidence of fewer than three cases per million individuals annually, has posed significant challenges in identifying reliable prognostic markers (22). Existing studies are often limited by outdated case data, insufficient adjustment for critical variables, and reliance on poorly validated models. Consequently, findings from these studies frequently conflict, underscoring the need for more rigorous and representative research methodologies. Given the uniformly poor outcomes associated with ES, there is no globally recognized risk classification system for patients to date. To address these challenges, we employed a nomogram (a robust prognostic model) that incorporates multiple variables to estimate individual survival probabilities. This model was rigorously validated using an independent dataset, with variables carefully selected based on prior research to ensure accuracy and relevance. Addressing these gaps necessitates an in-depth understanding of tumor-immune dynamics and the identification of reliable therapeutic targets.

To unravel these tumor-immune interactions, we integrated single-cell and bulk RNA sequencing data to explore cellular communication between immune and tumor cells in ES. Prognostic factors were identified through univariate and multivariate Cox proportional hazard analyses using T cell-specific genes, leading to the discovery of three independent prognostic markers: BDP1, CLEC11A, and ID3. These markers were used to construct a nomogram for predicting 3- and 5-year overall survival, providing a valuable tool for clinical decision-making in ES management. GO analysis revealed enrichment in biological processes such as positive regulation of cell organization, synapse organization, neuron projection organization, modulation of chemical synaptic transmission, regulation of cell cycle activity, and macrophage-mediated differentiation. These processes collectively highlight the multifaceted nature of cancer progression in ES. Further, KEGG enrichment analysis demonstrated that the identified marker genes are predominantly involved in key oncogenic pathways, including the PI3K-Akt signaling pathway, focal adhesion, and regulation of the actin cytoskeleton. These pathways play crucial roles in the pathogenesis of ES (23–25), suggesting that targeting them could enhance the effectiveness of immunotherapeutic strategies. Like most sarcomas, ES is categorized as an immunologically “cold” tumor due to its low TMB and limited immunogenicity (26). However, our immune infiltration analysis suggests that despite its “cold” classification, ES can elicit immune responses, as evidenced by increased infiltration of diverse lymphocyte populations, including memory and effector T cells, natural killer (NK) cells, and dendritic cells (DCs). These findings will open new avenues for leveraging immunotherapies in ES, potentially overcoming the challenges associated with its low immunogenicity.

Nomograms have emerged as powerful statistical tools for predicting patient outcomes across various cancers. By integrating multiple clinical or molecular variables, nomograms enable individualized risk stratification and often surpass traditional stage-based systems in predictive accuracy. Their application mitigates subjective bias and offers clinical guidance, particularly in cases where the potential benefit of additional treatment remains uncertain (27–30).

In recent years, the prognostic value of nomograms has gained momentum in sarcoma research, including Ewing Sarcoma (ES). For instance, Hsu et al. developed a nomogram for adult ES patients based on SEER data, incorporating age, surgery, chemotherapy, and TNM stage, with AUC values of 76.4, 77.3, and 76.6 for predicting 3-, 5-, and 10-year overall survival (OS), respectively (31). Similarly, Wen et al. proposed a multicenter prognostic model for ES combining age, bone metastasis, tumor size, and chemotherapy, demonstrating strong discrimination and calibration (32).

Extending these findings, our study introduced and validated a gene-based nomogram constructed through integrated single-cell and bulk RNA-seq analyses. This model demonstrated high predictive performance, with AUC values of 0.85, 0.82, and 0.78 for 1-, 3-, and 5-year OS, respectively, offering both prognostic value and biological insight.

Furthermore, to determine whether the immune clusters were associated with patient prognosis, we examined the correlation between the risk score and the immune subtypes C1 and C2 (**Supplementary Figure 3**). The analysis revealed no statistically significant association, indicating that the immune classification and the prognostic model represent independent dimensions of tumor characterization. While the immune clusters reflect variations in the tumor immune microenvironment, the risk score stratifies patients based on survival-related gene expression. These findings highlight the distinct roles of each approach and support their separate use in immune profiling and prognostic evaluation.

We then checked the *ID3* risk gene role in ES progression *in vitro*. The ID family of proteins (ID1–4), which interact with basic helix-loop-helix (bHLH) transcription factors to inhibit bHLH-mediated transcription, are key regulators of differentiation and chemoresistance in cancer cells originating from diverse cellular lineages (33–43). Among them, ID1 and ID3, like ID2, are rapidly degraded via the proteasome and function as oncogenes in certain tumor types (33, 40). Additionally, ID proteins are frequently co-expressed and exhibit overlapping roles, particularly in processes related to differentiation and development (33). In ES, cell lines and primary tumors have been shown to exhibit elevated levels of ID2, with evidence that EWS-FLI1 binds to the ID2 promoter to upregulate its transcription (44–48). Similarly, ID3 is overexpressed in ES, and it has been hypothesized that this upregulation contributes to ES progression. To explore this, we investigated downstream genes of ID3, including *P21*, *ASCL1*, *TCF3*, *SIRPA*, *SIX1*, *THRA*, and *SOX8*, in ES cell lines. The results demonstrated that ID3 knockout led to the upregulation of *P21*, *TCF3*, and *THRA*. The *P21* gene encodes a protein that regulates the cell cycle and has additional functions beyond CDK activity regulation. It has been previously identified as a transcriptional target of *P53* (49–53). In the context of ES, prior studies have indicated that *P21* may be a direct transcriptional target of EWS-FLI, although the *in vivo* binding site remains elusive (54). Transcription factor 3 (*TCF3*), which encodes a protein involved in transcriptional activation and lymphocyte development and differentiation, has been reported to exhibit low expression in ES, correlating with poor survival outcomes (55).

To sum up, our study highlighted that T cell modulation and the development of a robust prognostic model are essential for improving patient outcomes in ES. Our model, integrating immune and molecular markers, provides a reliable tool for predicting patient survival. ID3, in particular, plays a crucial role in immune evasion and tumor progression. Its overexpression, influencing key genes like *P21, TCF3*, and *THRA*, highlighting *ID3* as a promising target for future immunotherapies, offering the potential for more effective treatment strategies in ES.

## Data Availability

The original contributions presented in the study are included in the article/**Supplementary Material**. Further inquiries can be directed to the corresponding authors.
