# Supplementary material for: Integrative analysis of T cell-associated markers in Ewing sarcoma reveals prognostic signatures and immune dynamics
Source: Front Immunol. 2025 Jun 18;16:1586544. doi: 10.3389/fimmu.2025.1586544 (PMC12213708; doi:10.3389/fimmu.2025.1586544)
Supplement: Supplementary file 1 [file DataSheet1.zip › Supplementary Figures.DOCX]

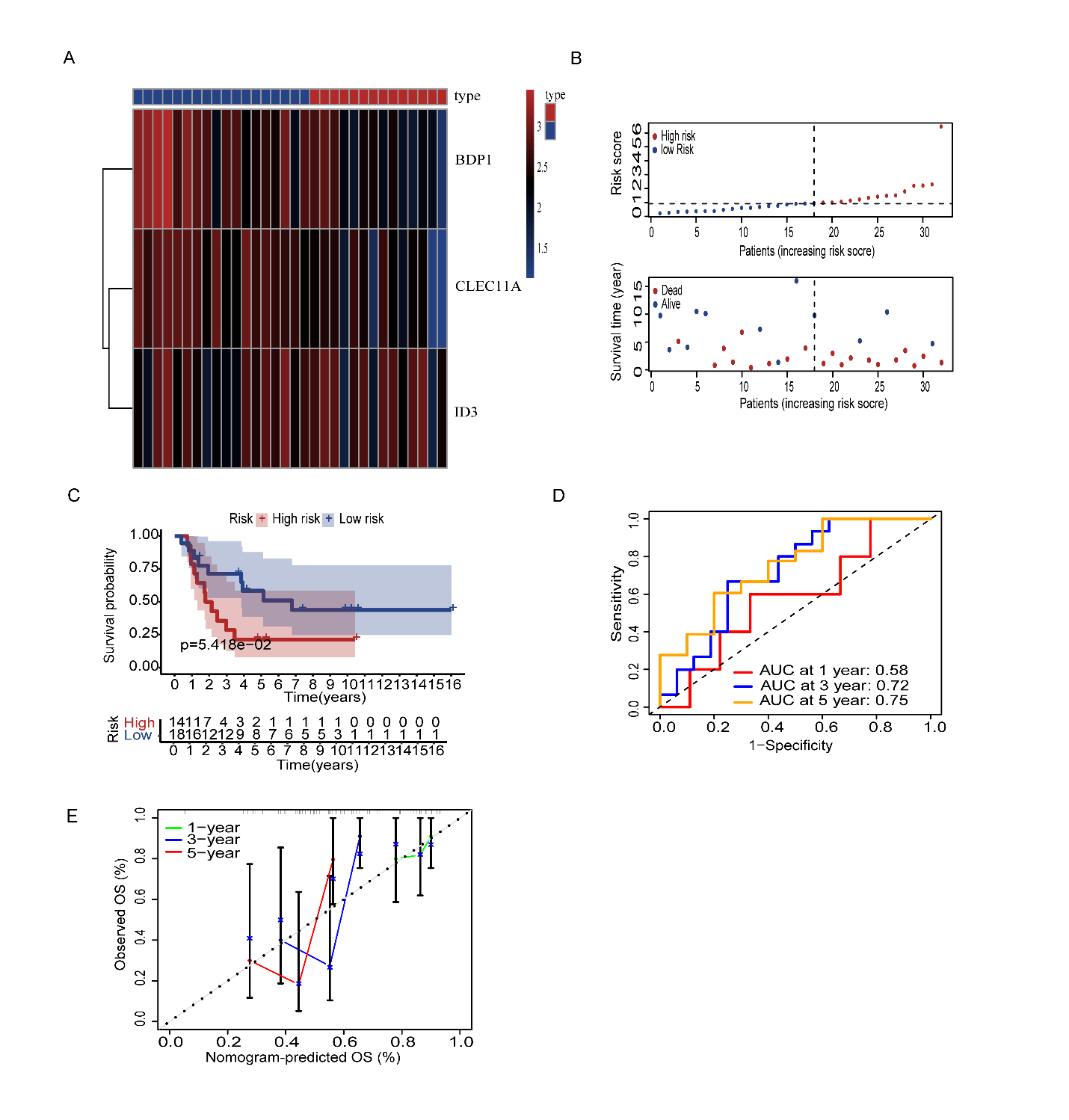


**Supplementary Figure S1.** Risk stratification and survival analysis. (A) Risk score distribution across patients, stratified into high-risk and low-risk groups. (B) Heatmap of prognostic signature genes. (C) Kaplan-Meier survival curves showing significantly worse survival in the high-risk group (p = 5.418e-02). (D) ROC curve evaluating the predictive accuracy of the risk score model. (E) Calibration plot comparing nomogram-predicted overall survival (OS) with observed OS, demonstrating good agreement.


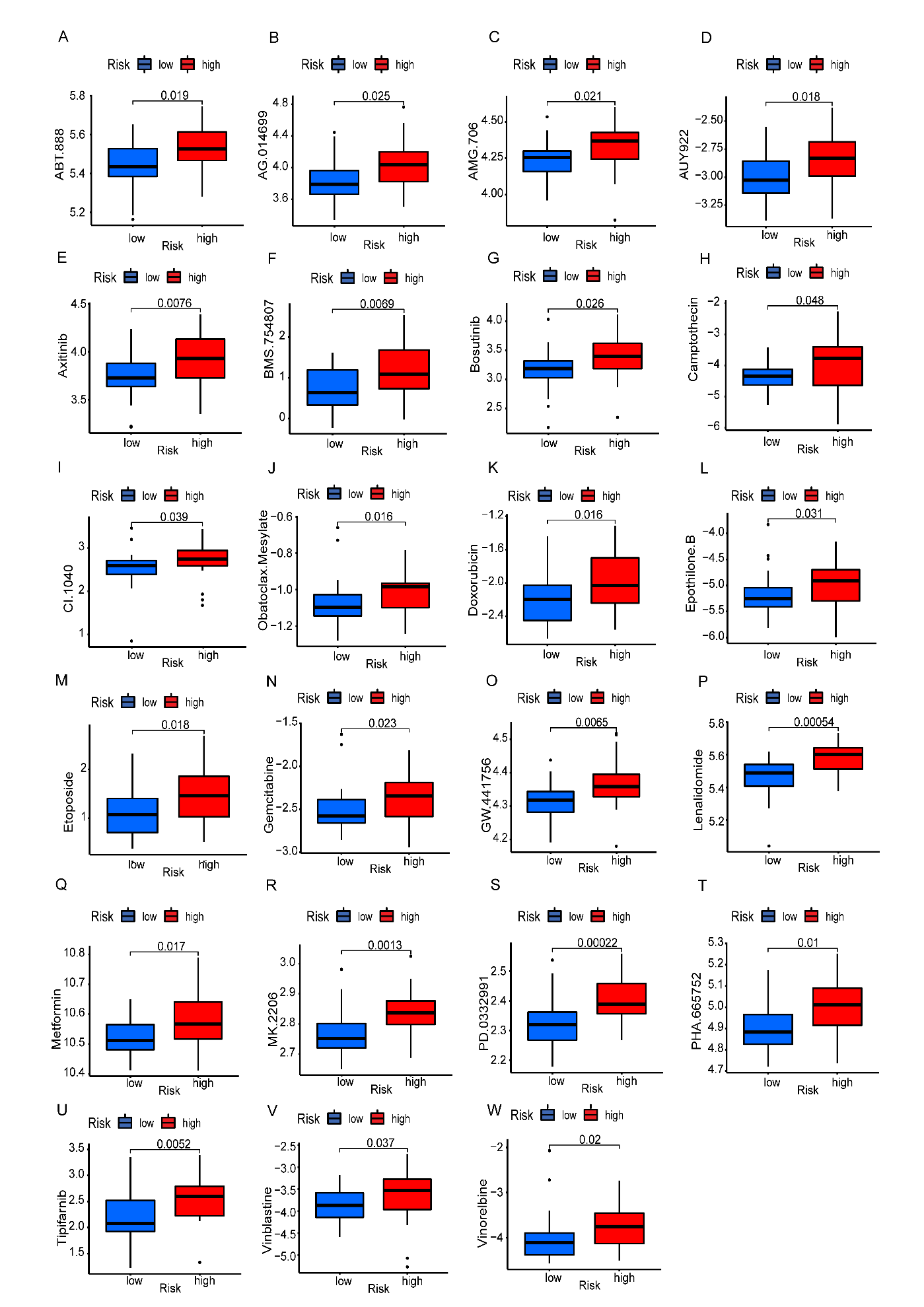


**Supplementary Figure S2.** Evaluation of drug sensitivity. Box plot displays the Comparison of drug responses between high-risk and low-risk groups with statistical significance.


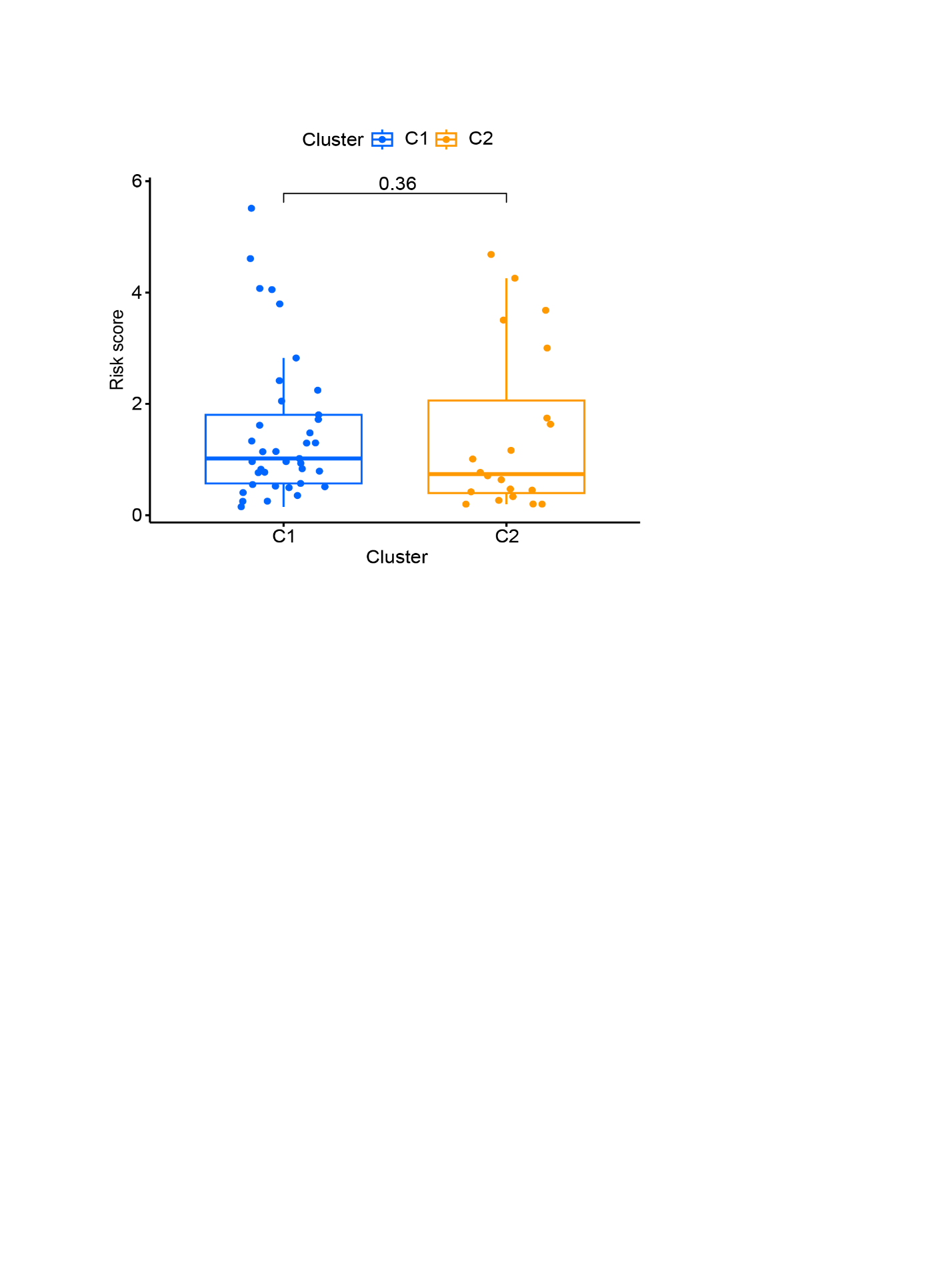


**Supplementary Figure S3.** Comparison of risk scores between immune clusters C1 and C2. No significant difference was observed, indicating independence between immune clustering and prognostic risk stratification.
